# Characteristics, treatment and quality of life of stable coronary artery disease patients with or without angina: Insights from the START study

**DOI:** 10.1371/journal.pone.0199770

**Published:** 2018-07-12

**Authors:** Leonardo De Luca, Pier Luigi Temporelli, Donata Lucci, Furio Colivicchi, Paolo Calabrò, Carmine Riccio, Antonio Amico, Franco Mascia, Emanuele Proia, Andrea Di Lenarda, Michele Massimo Gulizia, on behalf of the START Investigators

**Affiliations:** 1 Division of Cardiology, S. Giovanni Evangelista Hospital, Tivoli (Roma), Italy; 2 Division of Cardiology, Istituti Clinici Scientifici Maugeri, IRCCS, Veruno (Novara), Italy; 3 ANMCO Research Center, Firenze, Italy; 4 Division of Cardiology, S. Filippo Neri Hospital, Roma, Italy; 5 Department of Cardiology, Azienda Ospedaliera Sant'Anna e San Sebastiano, Caserta, Italy; 6 Division of Cardiology, S. Giuseppe Hospital, Copertino (Lecce), Italy; 7 Division of Cardiology Ave Gratia Plena Hospital, Piedimonte Matese (Caserta) Italy; 8 Cardiology, Azienda Sanitaria Universitaria Integrata di Trieste, Trieste, Italy; 9 Division of Cardiology, Garibaldi-Nesima Hospital, Catania, Italy; University of Messina, ITALY

## Abstract

Data on contemporary management patterns of angina in patients with stable coronary artery disease (CAD) are scarce. We sought to describe the current presentation, management, and quality of life of stable CAD patients with or without angina, using the data from the START (STable Coronary Artery Diseases RegisTry) study. START was a prospective, observational, nationwide study aimed to evaluate the presentation, management, treatment and quality of life of stable CAD presenting to cardiologists during outpatient visits or discharged from cardiology wards. Among the 5070 consecutive stable CAD patients enrolled in 183 participating centers over a 3-month period, 3714 (73.2%) had no angina and 1356 (26.8%) presented with angina. Patients with angina underwent more frequently coronary angiography (92.7% vs 84.9%; p<0.0001) and other diagnostic imaging procedures compared to those without angina. In addition, patients with angina received more frequently different combinations of first line therapies and angina relief drugs compared to patients without angina. The quality of life, assessed with the EQ 5D-5L questionnaire, did not differ between the two groups, with the exception of the ‘pain or worry’ domain that was higher in patients with compared to those without angina (p<0.0001). Current management and treatment of stable CAD patients with angina is still suboptimal and different compared to those without angina. Our findings highlight the need for disseminating best-practice patterns and improving guidelines adherence for the management of angina even among cardiologists.

## Introduction

Despite marked improvements in the management of stable coronary artery disease (CAD) during the last decades, the burden of this disease remains the single most important cause of morbidity and mortality in the western world [1,2]. Angina is the initial clinical manifestation in many patients who present with CAD, and it is associated with worse clinical outcomes regardless of the presence of myocardial ischemia on noninvasive testing [3–5]. Current guidelines recommend that establishing a diagnosis of stable angina should be pursued in parallel with managing symptoms and initiating preventive therapies.

Although therapeutic options for the management of angina have evolved over time, contemporary data on burden and management patterns of angina in patients presenting to cardiologists is limited. We sought to describe the current presentation, management, and quality of life of stable CAD patients with or without angina, using the data from the START (STable Coronary Artery Diseases RegisTry) study [6].

## Methods

The design and main results of the START registry have been published previously (6). Briefly, the START was a prospective, observational, nationwide study aimed to evaluate the current presentation, management, treatment and quality of life of stable CAD patients as seen by cardiologists in clinical practice in Italy, during a 3-month period [6]. Patients with stable CAD presenting to a cardiologist during an outpatient visit or those discharged from a cardiology ward were eligible if they had at least 1 of the following clinical conditions: 1) typical stable angina and/or non-anginal symptoms [1,2]; 2) documented ischemia at stress test with or without symptoms; 3) previous revascularization, such as percutaneous coronary intervention (PCI) or coronary bypass grafting (CABG); 4) prior episode (occurred at least 30 days from enrolment) of acute coronary syndrome (ACS); 5) elective admission for coronary revascularization (including staged procedures). We excluded patients aged <18 years old, those with Canadian Cardiovascular Society (CCS) IV angina or with atypical chest pain that in all probability was not related to CAD [6]. Enrolment was made at the end of outpatient or day-hospital visit or at hospital discharge. In the present analysis we analyze the data of patients presenting with or without angina, defined as at least one episode of typical angina at rest occurring in the last 3 months from enrolment.

The Italian Association of Hospital Cardiologists (ANMCO) invited to participate all Italian cardiology wards, including university teaching hospitals, general and regional hospitals, and private clinics receiving stable CAD patients. No specific protocols or recommendations for evaluation, management, and/or treatment have been put forth during this observational study. However, current guidelines for the management of patients with stable CAD have been discussed during the investigator meetings.

All patients were informed of the nature and aims of the study and asked to sign an informed consent for the anonymous management of their individual data. The local Institutional Review Board (IRB) of the coordinating center (Garibaldi-Nesima Hospital, Catania) initially approved the study protocol. Subsequently all local IRB of the participating centers (see appendix) approved the study, according to the current Italian rules.

One-hundred eighty-three cardiology centers included consecutive patients in the survey in different periods of 3 months between March 2016 and February 2017 [6].

### Data collection and data quality

Data on baseline characteristics, including demographics, risk factors and medical history, were collected. Information on the use of diagnostic cardiac procedures, type and timing of revascularization therapy (if performed), and use of pharmacological or non-pharmacological therapies were recorded on an electronic case report form (CRF).

Optimal medical therapy (OMT) was defined as patients being prescribed aspirin or thienopiridine, β-blocker, and a statin, at any dosage. To be categorized as receiving OMT, individual patients must have been either prescribed or had reported contraindications to all medications in each category. The composite OMT percentages were calculated using the number of patients receiving OMT, as defined above, as the numerator and the total number of patients in the entire cohort as the denominator. Data on the use of angiotensin-converting enzyme inhibitors (ACE-I) or angiotensin II receptor blockers (ARBs) were recorded and could be used to calculate their use among those patients in whom they were clinically indicated. Therefore, given that the guidelines for stable CAD recommend an ACE inhibitor or ARB for some subgroups of patients [1,2], we also examined OMT, defined as patients being prescribed aspirin or thienopyridine, β-blocker, statin, and ACE inhibitor or ARB, if indicated by an ejection fraction of less than 40%, hypertension, diabetes, or chronic renal dysfunction (eligible patients).

All patients in the study were also asked to complete the self-administered EuroQoL 5D-5L quality of life questionnaire [7]. The EQ 5D-5L is a simple, generic health-related quality of life instrument comprising a visual analogue scale (VAS) of self-rated general health and 5 domains (mobility, self-care, daily activities, pain/worry, and anxiety/depression).

Data were collected using a web-based, electronic CRF with the central database located at the ANMCO Research Center. By using a validation plan, integrated in the data entry software, data were checked for missing or contradictory entries and values out of the normal range.

### Statistical analysis

The study cohort was stratified according to the presence or absence of angina. Categorical variables are presented as number and percentages and compared by the chi-squared test. Continuous variables are presented as mean and standard deviation (SD), except for timing from diagnostic procedures to enrolment, triglycerides and health status assessment, which are reported as median and interquartile range (IQR). Continuous variables were compared by the t test, if normally distributed, or by the Mann-Whitney U test, if not. A p value < 0.05 was considered statistically significant. All tests were 2-sided. Analyses were performed with SAS system software, version 9.2.

## Results

Among the 5070 consecutive stable CAD patients enrolled, 3714 (73,2%) had no angina and 1356 (26.8%) presented with angina. The majority of symptomatic patients (93.0%) had mild to moderate angina (CCS class I or II).

Baseline characteristics of patients with and without angina are shown in Table 1. Patients with angina were older, with a higher incidence of risk factors such as diabetes mellitus, hypertension and peripheral artery disease compared to patients not presenting angina (Table 1). On the other hand, patients without angina presented more frequently a history of heart failure or atrial fibrillation, prior ACS, previous revascularization and chronic renal dysfunction.

**Table 1:** Baseline clinical, hemodynamic and laboratory variables of patients without and with angina.

	Without Angina n = 3714	With Angina n = 1356	P value
Age (years), mean±SD	67.3±10.9	68.3±9.5	0.002
Age >75 years, n (%)	948 (25.5)	335 (24.7)	0.55
Females, n (%)	713 (19.2)	295 (21.8)	0.04
BMI (kg/m^2^), mean±SD	27.4±4.0	27.3±3.9	0.56
Active smokers, n (%)	675 (18.2)	212 (15.6)	0.04
Hypercholesterolemia, n (%)	2767 (74.5)	1023 (75.4)	0.50
Diabetes mellitus, n (%)	1092 (29.4)	466 (34.4)	0.0007
Hypertension, n (%)	2890 (77.8)	1134 (83.6)	<0.0001
History of Atrial Fibrillation, n (%)	548 (14.8)	147 (10.8)	0.0003
Chronic renal dysfunction, n (%)	475 (12.8)	127 (9.4)	0.0009
Peripheral artery disease, n (%)	292 (7.9)	159 (11.7)	<0.0001
COPD, n (%)	458 (12.3)	145 (10.7)	0.11
Sleep apnea, n (%)	128 (3.5)	35 (2.6)	0.12
Malignancy, n (%)	237 (6.4)	94 (6.9)	0.48
Depression, n (%)	422 (11.4)	109 (8.0)	0.0006
Previous stroke/TIA, n (%)	195 (5.3)	81 (6.0)	0.32
History of major bleeding events, n (%)	72 (1.9)	23 (1.7)	0.57
History of heart failure, n (%)	562 (15.1)	118 (8.7)	<0.0001
Prior ACS, n (%)	2980 (80.2)	444 (32.7)	<0.0001
Previous revascularization, n (%)	3189 (85.9)	777 (57.3)	<0.0001
Ejection fraction (%), mean±SD *available for 4599 (90*.*7%) pts*	53.3±10.3	55.7±8.7	<0.0001
SBP (mmHg), mean±SD	129.6±16.5	131.4±16.3	0.0004
HR (bpm), mean±SD	66.0±10.9	65.4±10.6	0.09
Atrial fibrillation at entry ECG, n (%)	163 (4.4)	48 (3.5)	0.18
Hb (gr/dl), mean±SD *available for 4139 (81*.*6%) pts*	13.6±1.8	13.6±1.6	0.34
Creatinine (mg/dl), mean±SD *available for 4130 (81*.*5%) pts*	1.09±0.57	1.04±0.56	0.003
Total cholesterol (mg/dl), mean±SD *available for 3669 (72*.*4%) pts*	152.2±38.4	161.2±40.0	<0.0001
LDL cholesterol (mg/dl), mean±SD *available for 3210 (63*.*3%) pts*	85.8±33.7	91.4±34.0	<0.0001
Triglycerides (mg/dl), median (IQR) *available for 3557 (70*.*2%) pts*	110 (83–149)	116 (86–164)	0.002
Glycemia (mg/dl), mean±SD *available for 3778 (74*.*5%) pts*	113.8±36.0	113.6±36.5	0.89

ACS: acute coronary syndrome (STEMI or NSTE-ACS); BMI: body mass index; COPD: chronic obstructive pulmonary disease; ECG: electrocardiogram; Hb: hemoglobin; HR: heart rate; LDL: low density lipoprotein; SBP: systolic blood pressure; TIA: transient ischemic attack.

### Diagnostic procedures, lifestyle and pharmacological management

The vast majority of stable CAD patients had received a coronary angiography (92.7% with vs 84.9% without angina; p<0.0001), followed by transthoracic echocardiography (80.4% vs 86.6%; p<0.0001), stress test (43,6% vs 30.6%; p<0.0001), Holter ECG (9.2% vs 14.8%; p<0.0001), myocardial scintigraphy (24.3% vs 7.9%; p<0.0001), stress echocardiography (7.3% vs 3.7%; p<0.0001), coronary computed tomography angiography (6.0% vs 1.0%; p<0.0001) and cardiac MRI (1.3% vs 0.4%; p = 0.005). Time intervals between imaging procedures and enrolment are depicted in Table 2. In general, patients with angina underwent coronary angiography and other diagnostic imaging procedures earlier compared to patients without angina.

**Table 2:** Timing (days) from diagnostic procedures to enrollment, median (IQR).

	Without Angina n = 3714	With Angina n = 1356	P value
Coronary angiography	280 (65–1256)	2 (1–99)	<0.0001
Transthoracic echocardiogram	38 (1–175)	8 (1–78)	<0.0001
Stress test	51 (1–309)	36 (11–106)	0.26
Holter ECG	46 (10–256)	52 (6–315)	0.83
Myocardial perfusion scintigraphy	359 (116–1106)	63 (31–227)	<0.0001
Stress echocardiography	134 (7–675)	65 (12–344)	0.33
CCTA	120 (50–364)	30 (15–198)	0.006
Cardiac MRI	736 (93–982)	103 (7–199)	0.06

CCTA: Coronary computed tomography angiography; ECG: electrocardiogram; IQR: interquartile range; MRI: Magnetic Resonance Imaging.

A personalized diet was prescribed in 56.8% of patients with angina vs 58.9% of those without angina (p = 0.18), physical activity programs were suggested in 59.1% vs 67.3% (p<0.0001) and a smoking cessation was recommended in 68.9% vs 71.6% (p = 0.45) of currently smokers patients, respectively.

**Fig 1:**
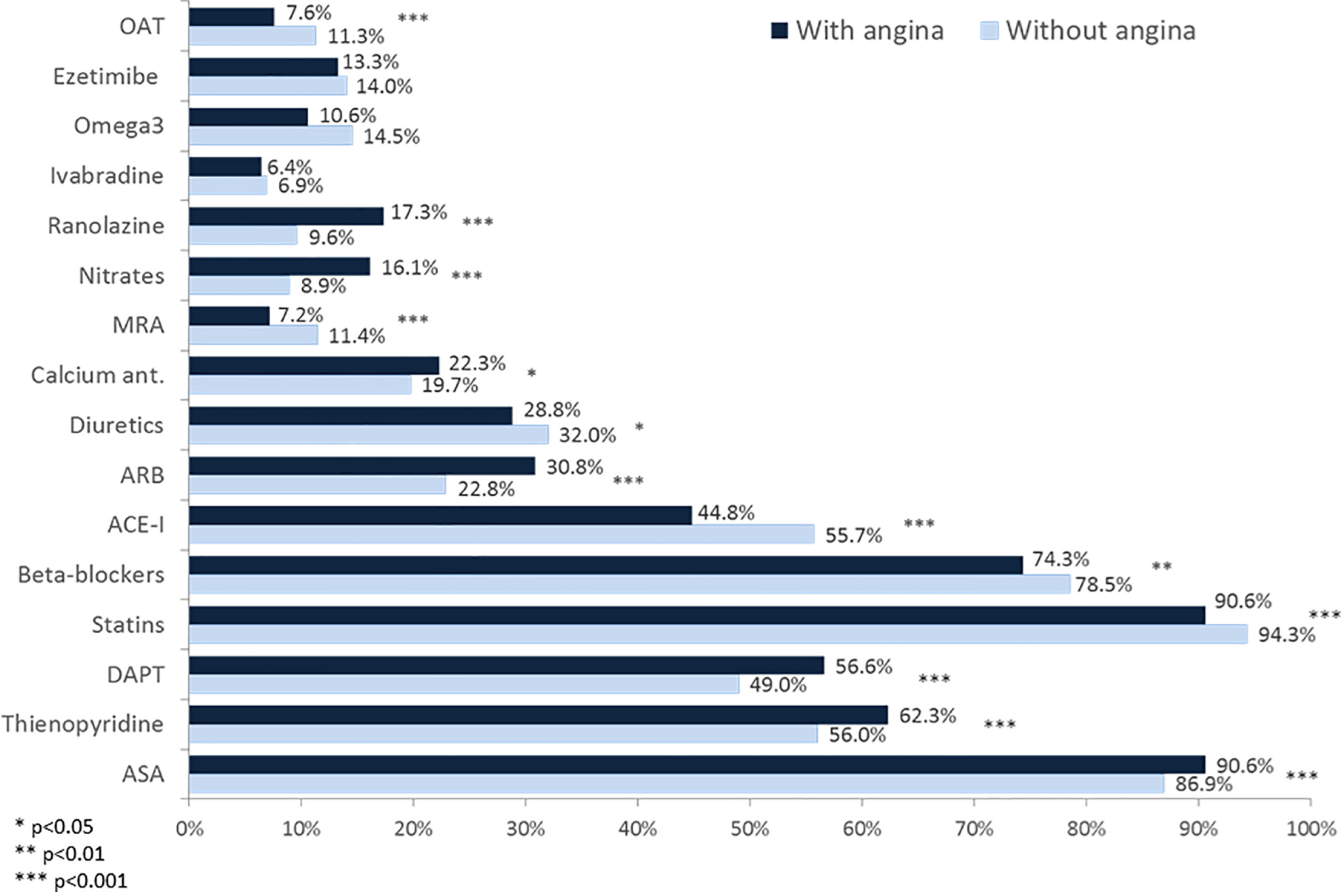
Cardiovascular drugs^†^ prescribed at the end of the visit or at hospital discharge in patients with and without angina. ^†^Drugs not reported have been used in less than 5% of cases. ACE-I: angiotensin converting enzyme inhibitors; ARB: angiotensin receptor blockers; ASA: acetylsalicylic acid; DAPT: dual antiplatelet therapy; MRA: mineralocorticoid receptor antagonist; OAT: oral anticoagulation therapy.

**Fig 2:**
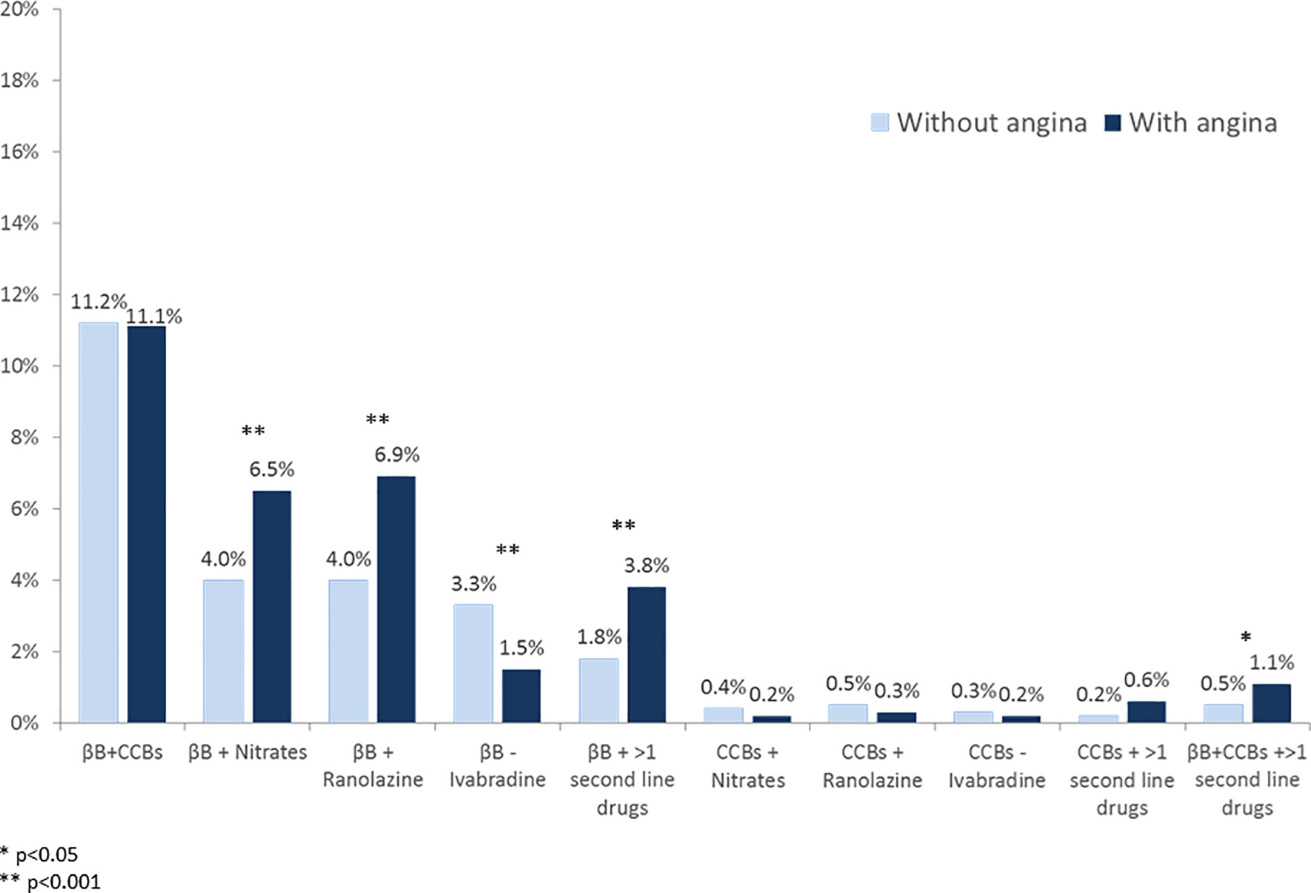
Use of combinations of angina relief drugs in patients with and without angina. βB: beta-blockers; CCBs: calcium channel blockers.

Pharmacological therapies prescribed at the end of the visit or at discharge in the two groups are depicted in Fig 1. Statins, beta-blockers, ACE-I, diuretics, omega3, MRAs and oral anticoagulants were prescribed more frequently in stable CAD patients without angina, while patients with angina received more frequently antiplatelet agents and drugs for angina relief. Patients with angina also received more often different combinations of first line therapies and angina relief drugs compared to patients without angina (Fig 2).

In general, patients with angina received less frequently OMT (aspirin or thienopyridine, β-blocker, and statin) in the entire cohort (67.0% vs 70.1%, p = 0.03), and OMT (aspirin or thienopyridine, β-blockers, statin, and ACE-I or ARB) in eligible patients (54.4% vs 58.5%, p = 0.02), compared to those without angina.

### Quality of life assessment

The EQ 5D-5L questionnaire was completed by 1308 (96.5%) patients with angina and 3545 (95.5%) without angina (p = 0.12). The median (IQR) VAS score of self-rated general health status was 75 (60–85) in both groups (p = 0.51).

**Fig 3:**
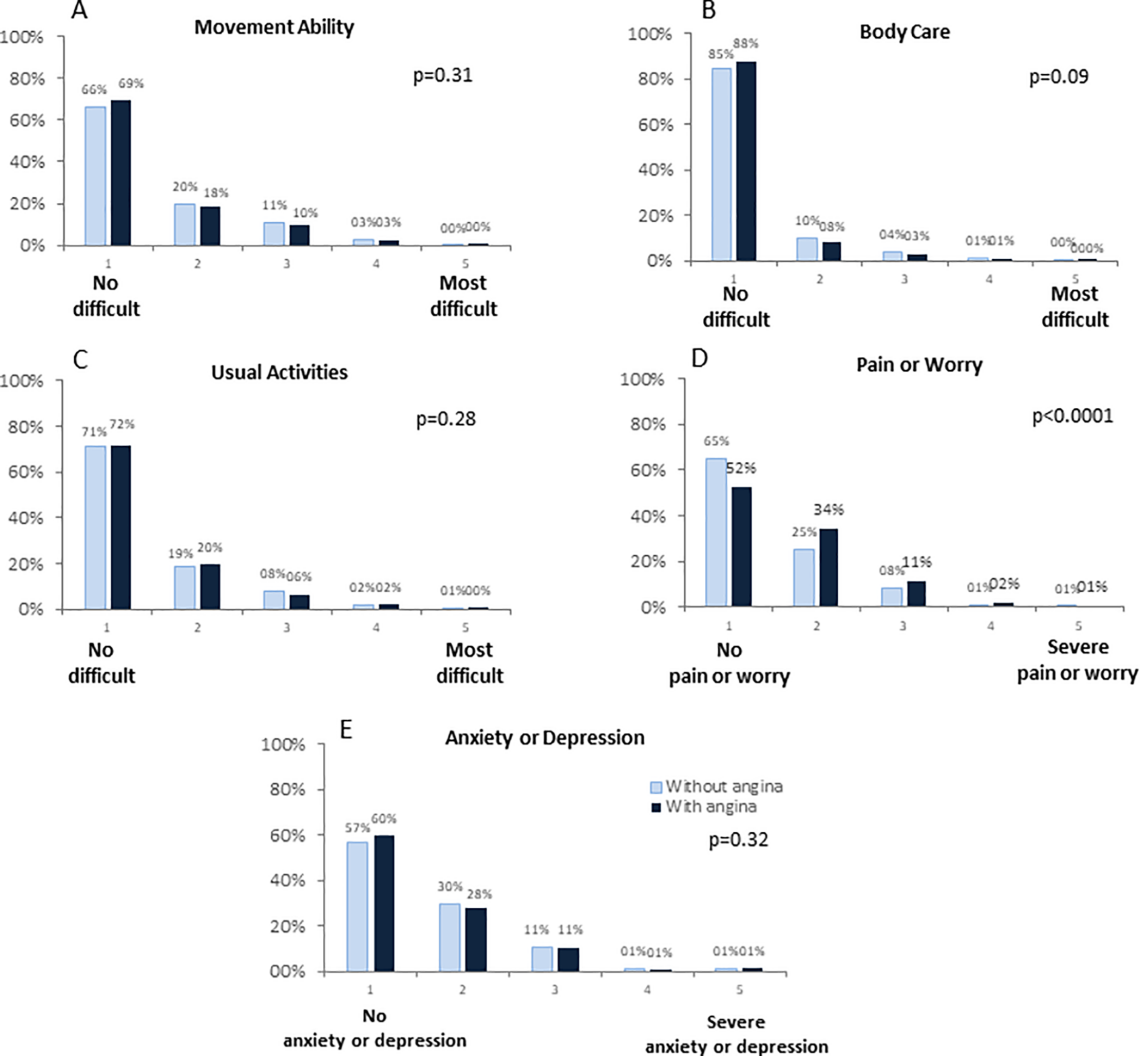
Domain of movement ability (panel A), body care (panel B), daily activities (panel C), pain/worry (panel D), and anxiety/depression (panel E) of the EQ 5D-5L questionnaire in patients with and without angina.

Over 65% of patients reported as “no problems” in all EQ-5D-5L domains (66.1–87.8%), except for pain and worry that, as expected, was present, with different degrees, in around 48% of patient with angina and depression/anxiety that was present in at least 42% of patients enrolled. The single domains with the 5 levels of severity on each domain are represented in Fig 3 and, with the exception of pain or worry, did not differ between the 2 groups.

## Discussion

Angina is the initial manifestation in approximately 25–50% of all patients who present with CAD, with wide variability across surveys [3,4,8–10] which may reflect differences in the recognition and management by provider type (e.g., cardiologist vs general practitioner) or by country (eg, more frequent use of revascularization in western countries). Even in our registry, patients with angina encountered the 27% of the overall population and presented a more severe risk profile compared to patients without angina. Indeed, chronic angina is associated with considerable rates of adverse cardiovascular events, especially if treated suboptimally [11–13]. For many patients, aggressive pharmacologic intervention is necessary in order to alleviate anginal symptoms [1,2]. Some anti-ischemic compounds, such as drugs for heart rate reduction and metabolic compounds, together with lifestyle changes, regular exercise training, patient education and myocardial revascularization play an important role in reducing or removing symptoms over the long term [1,2]. Current guidelines recommend a multifaceted therapeutic approach for patients with stable angina with the double aim of preventing cardiovascular events and reducing angina [1,2]. In the present survey, more than 90% of patients with angina received aspirin and statins at the end of the visit or at hospital discharge, while drugs for angina relief were prescribed in a minority of patients, with the exception of beta-blockers used in approximately 74% of cases. The rate of pharmacological therapies used in patients enrolled in the START registry is closer to that of contemporary registries on stable CAD, such as the chronic ischemic cardiovascular disease (CICD)-Pilot survey, an european observational longitudinal registry in CAD and/or PAD patients [4], and the CLARIFY registry, a large international registry of patients with stable angina and/or documented ischemia [5].

Beta-blockers are recommended as first-line treatment for angina relief. However, beta blockers have not been demonstrated to be more effective than other anti-anginal drugs on myocardial ischemia for stable angina patients [1,2]. Among the pharmacological agents recently introduced for the management of chronic stable angina, ranolazine and ivabradine represent two innovations. In fact, even if both drugs act by reducing myocardial work and thus oxygen consumption, this happens by a peculiar mechanism unlike that of conventional antischemic drugs. A body of evidence found that two drugs are useful in ischemic patients both at rest or during exercise [14,15]. Thus, the two drugs represent an adjunctive therapeutic modality for the treatment of chronic stable angina, especially when conventional antianginal drugs are insufficient or inadequate. In our series, patients with angina received more frequently antianginal drugs alone and in combination with beta-blockers or other first line therapies compared to patients without angina. However, the combinations of angina relief drugs were rarely employed, suggesting the use of these therapies can still be improved in clinical practice, especially before relying on invasive coronary angiography and revascularization procedures, as suggested by current guidelines [1,2]. In addition, patients with angina less frequently received OMT or lifestyle modification programs compared to patients without angina. These findings suggest that important opportunities to increase guidelines adherence still exist in stable CAD. Indeed, current guidelines recommend to implement medical therapy regardless revascularization, based on results of the large COURAGE (Clinical Outcomes Utilizing Revascularization and Aggressive Drug Evaluation) trial [16,17] and several meta-analyses [18–20] demonstrating there is no benefit of PCI compared to OMT in preventing myocardial infarction or death in stable CAD patients.

Coronary angiography is a gold standard test in the evaluation of severity of CAD and it is indicated in patients with a high pretest probability of CAD and in symptomatic patients with inconclusive initial noninvasive tests. In our study, coronary angiography was widely employed, especially when compared to other registries previously conducted in European countries [3,4]. In particular, patients with angina underwent more frequently and earlier coronary angiography and other diagnostic imaging procedures compared to patients without angina. A large international trial, the International Study of Comparative Health Effectiveness With Medical and Invasive Approaches (clinicaltrials.gov identifier NCT01471522), is exploring whether angiography with a view to revascularization in addition to optimal medical management is superior to optimal medical management alone in patients with myocardial ischemia. In this regard, the ORBITA trial recently randomized 200 patients with single-vessel stenoses to either PCI or placebo using a blinded sham procedure showing no significant difference in the primary endpoint of exercise time increment between arms [21]. These findings further questioned the benefits of percutaneous revascularization in the setting of stable angina.

### Study limitations

Our study must be evaluated in the light of some limitations. First, some important information for the assessment of long-term risk of cardiovascular events in stable CAD, such as the frequency and duration of angina, are missing and no standardized evaluation of angina was used. Second, the data reported in the present analysis are limited to the time of the visit or hospitalization period and we do not have data on long-term persistence to prescribed therapies and relative outcomes. However, a clinical follow-up at 1 year from enrolment is ongoing. Finally, even if the participating centers were asked to include in the registry all consecutive patients admitted with stable CAD, we were not able to verify the enrolment process, due to the absence of administrative auditing. We believe that it is unlikely however that selective enrolment in few sites may have substantially changed the study results.

## Conclusions

Using data from a nationwide, contemporary, prospective registry of consecutive unselected stable CAD patients enrolled in 183 cardiology Italian centers, we describe different management and treatment patterns of stable CAD patients with mild to moderate angina compared to patients without angina presenting to a cardiologist. Indeed, patents with angina more frequently undergo coronary angiography and some non-invasive diagnostic procedures and receive more drugs for angina relief, alone or in combination with first-line therapies, as compared to patients without angina. Our findings highlight the need for further work on defining and disseminating best-practice patterns and improving guidelines adherence for the management of angina even among cardiologists.

### Steering committee

L De Luca (Chairman, lead author of the START Registry), MM Gulizia (co-chairman), PL Temporelli, C Riccio, F Colivicchi, AF Amico, D Formigli, G Geraci, A Di Lenarda.

### Executive committee

L De Luca, AP Maggioni, D Lucci.

### Coordinating center

ANMCO Research Center (AP Maggioni, D Lucci, A Lorimer, G Orsini, L Gonzini, G Fabbri, P Priami).

### Participating centers and investigators

Trieste, Maggiore (P Maras, F Ramani); Pavia, Istituto di Cura Città di Pavia (C Falcone, I Passarelli, S Mauri); Napoli, AORN Colli-Monaldi, UOC Cardiologia-SUN (P Calabrò, R Bianchi, G Di Palma); Caserta, AO S. Anna e S. Sebastiano, UO Cardiologia-UTIC (F Mascia, A Vetrano, A Fusco); Piedimonte Matese (E Proia); Roma, San Filippo Neri (F Colivicchi, A Aiello); Roma, European Hospital (F Tomai, R Licitra, A Petrolini); Santa Maria Capua Vetere (B Bosco); Lecce, V. Fazzi, UO Cardiologia (F Magliari, M Callerame, T Mazzella); Vittoria (GV Lettica, G Coco, F Incao); Città di Castello (L Marinacci, S D'Addario); Sanremo (SN Tartaglione, S Ubaldi, FA Sanchez); Avola (P Costa, G Manca, M Failla); Benevento, AO G. Rummo (M Scherillo, V Procaccini, D Formigli); Bergamo, ASST Papa Giovanni XXIII (M Senni, EM Luminita); Cagliari, SS Trinità (P Bonomo, C Mossa, S Corda); Campobasso, Cardarelli (AR Colavita, G Trevisonno, G Vizzari); Cariati (N Cosentino, C Formaro); Corato (C Paolillo, IL Nalin); Cosenza, Annunziata (FM De Rosa, F Fontana, GF Fuscaldo); Cremona (E Passamonti, E Bertella, EV Calvaruso); Faenza (E Varani, F Tani, G Cicchitelli); Fermo (D Gabrielli, P Paoloni, A Marziali); Ferrara (G Campo, M Tebaldi, S Biscaglia); Foggia, Riuniti (M Di Biase, ND Brunetti, AM Gallotta); Gorizia (L Mattei, R Marini, F Balsemin); Magenta (M D'Urbano, R Naio, P Vicinelli); Massa, Apuane (G Arena, M Mazzini, N Gigli); Melito di Porto Salvo (B Miserrafiti, A Monopoli); Monza, Policlinico (A Mortara, P Delfino, MM Chioffi); Novara, AOU Maggiore della Carità, SCDU Clinica Cardiologica-Cardiologia I (P Marino, M Gravellone, L Barbieri); Palermo, AOR Villa Sofia-Cervello (A Ledda, G Geraci, MG Carmina); Pavia, IRCCS Policlinico San Matteo (AE Raisaro, C Di Giacomo, A Somaschini); Potenza, San Carlo, SSD Card. Riab. (ML Fasano, M Sannazzaro, R Arcieri); Reggio Emilia, S.M. Nuova (M Pantaleoni, C Leuzzi, G Gorlato); Roma, Santo Spirito (G Greco, A Chiera); Rozzano (TA Ammaturo, G Malanchini, MP Del Corral); Battipaglia (L Tedesco); Lecce, Casa di Cura Petrucciani (S Pede, LG Urso); Salerno (F Piscione, G Galasso); Varese, Circolo e Fond. Macchi (S Provasoli); Aversa (L Fattore, G Lucca); Grosseto (A Cresti); Caserta, AO S. Anna e S. Sebastiano, Cardiologia e Riabil. Cardiol. (A Cardillo); Pomezia (MS Fera, F Vennettilli); Roma, Umberto Primo, Cardiologia B—Cardiologia e Angiologia (C Gaudio, V Paravati); Bari, San Paolo (P Caldarola, N Locuratolo); Camposampiero (R Verlato, F De Conti); Conegliano (G Turiano, G Preti); Ascoli Piceno (L Moretti, S Silenzi); Lecce, V. Fazzi, UO Card. Interventistica-Emod. (G Colonna, A Picciolo); Ragusa (A Nicosia, C Cascone); Roma, Campus Biomedico (G Di Sciascio, F Mangiacapra); San Giovanni Rotondo (A Russo, M Villella); Carate Brianza (G Esposito); Cortona (F Cosmi, S D'Orazio); Jesi (C Costantini, A Lanari); Giugliano In Campania (P De Rosa, L Esposito); Arzignano (C Bilato, C Dalla Valle); Pavia, ICS Maugeri (M Ceresa, E Colombo); Reggio Calabria, Bianchi Melacrino Morelli (V Pennisi, G Casciola); Udine, Santa Maria Misericordia (M Driussi, T Bisceglia); Lumezzane (S Scalvini, F Rivadossi); Roma, Sant'Andrea (M Volpe, F Comito); Tradate, Galmarini (D Scorzoni, P Grimoldi); Cassano delle Murge (R Lagioia, D Santoro); Osio Sotto (N De Cesare, T Comotti); Legnano (A Poli, P Martina); Locri (MF Musolino, EI Multari); Feltre (G Bilardo, G Scalchi); Isernia (C Olivieri, F Caranci); San Vito al Tagliamento (D Pavan, G Ganci); Senigallia (A Mariani, E Falchetti); Avellino (T Lanzillo, A Caccavale); Novara, AOU Maggiore della Carità, Cardiologia II (AS Bongo, A Rizzi); Siena (R Favilli, S Maffei); Napoli, San Gennaro (M Mallardo, C Fulgione); Thiene (F Bordin); Trento, Santa Chiara (R Bonmassari, E Battaia); Troina (A Puzzo); Chioggia (G Vianello); Poggibonsi (A D'Arpino, M Romei); Albano Laziale, Albano-Genzano (G Pajes, S Petronzelli); Cesena (F Ghezzi); Monfalcone (S Brigido, L Pignatelli); Torino, Maria Pia Hospital (E Brscic, P Sori); Barletta (M Russo, E Biancolillo); Brindisi (G Ignone, NA De Giorgio); Formia (C Campaniello, P Ponticelli); Milano, San Raffaele (A Margonato, S Gerosa); Agrigento (A Cutaia, C Casalicchio); Andria (F Bartolomucci, C Larosa); Molfetta (T Spadafina, A Putignano); Orvieto (R De Cristofaro, L Bernardi); Viterbo (L Sommariva, A Celestini); Alessandria, Clinica Città di Alessandria (CM Bertucci, M Marchetti); Belluno (E Franceschini Grisolia, C Ammendolea); Casalmaggiore (M Carini); Fabriano (P Scipione, M Politano); Marsala (G Rubino, C Reina); Mormanno (N Peccerillo); Pescara (L Paloscia, A D'Alleva); Sarzana (R Petacchi); Aprilia (M Pignalosa, D Lucchetti); Boscotrecase (F Di Palma, RA La Mastra); Galatina (AF Amico, M De Filippis); Gavardo (B Fontanella, G Zanini); Lido di Camaiore (G Casolo, J Del Meglio); San Benedetto del Tronto, Madonna del Soccorso (VM Parato, E Genovesi); Somma Lombardo (A D'Alimonte, A Miglioranza); Latina, Polo Ospedaliero Integrato (N Alessandri, F Moscariello); Napoli, AORN Cardarelli (C Mauro, A Sasso); Napoli, AORN Colli-Monaldi, UOC Cardiologia (P Caso, C Petrillo); Teramo (C Napoletano, SR Paparoni); Rieti (V Bernardo, R Serdoz); Roccadaspide (R Rotunno, I Oppo); Taranto, Casa di Cura Villa Verde (A Aloisio, A Aurelio); Augusta (G Licciardello, L Cassaniti); Catania, Garibaldi-Nesima (MM Gulizia, GM Francese); Veruno (C Marcassa, PL Temporelli); Vigevano, Civile (R Villani, F Zorzoli); Polistena (F Mileto, M De Vecchis); Copertino (AF Amico, D Scolozzi); Genova, Padre Antero Micone (G Lupi, D Caruso); Palermo, Casa di Cura Candela (E Rebulla, B La Fata); San Bonifacio (M Anselmi, P Girardi); Alcamo (E Borruso, G Ferrantelli); Cento (B Sassone, S Bressan); Ciriè (M Capriolo, E Pelissero); Lugo (M Piancastelli, M Gobbi); Manduria (F Cocco, MG Bruno); Massa, FTGM—Stabilimento di Massa (S Berti, G Lo Surdo); Roma, San Camillo, Cardiologia 2—Ex Cardio 3 (P Tanzi, R De Rosa); Scorrano (E Vilei, MR De Iaco); Venezia (G Grassi, C Zanella); Castel Volturno (L Marullo, G Alfano); Lamezia Terme (P Pelaggi, R Talarico); Napoli, Loreto Mare (B Tuccillo, L Irace); Roma, Aurelia Hospital (F Proietti, G Di Croce); Sessa Aurunca (L Di Lorenzo, A Zarrilli); Imperia (M Bongini, A Ranise); Ivrea (A Aprile, C Fornengo); Melfi (V Capogrosso, A Tranghese); Napoli, Clinica Mediterranea (B Golia, A Marziano); Rovigo (L Roncon, C Picariello); Sassuolo (E Bagni, E Leci); Vallo della Lucania (G Gregorio, F Gatto); Frattamaggiore (F Piemonte, F Gervasio); Guastalla (A Navazio, E Guerri); Roma, Madre Giuseppina Vannini (E Belmonte, F Marino); Anzio (N Di Belardino, MR Di Nuzzo); Bari, Policlinico (M Epifani); Milano, San Carlo Borromeo (G Comolatti, B Conconi); Novara, Clinica San Gaudenzio (D Benea); Nuoro (G Casu, P Merella); San Giuseppe Vesuviano (MA Ammirati, VM Corrado); Civitanova Marche (D Spagnolo); Gallarate (SI Caico); Milano, Istituto Clinico Città Studi (S Bonizzato); Ravenna (M Margheri); Vercelli (L Corrado); Ancona, INRCA (R Antonicelli); Gela (C Ferrigno); Sant'Agata di Militello (A Merlino); Saronno (D Nassiacos); Sesto San Giovanni, IRCCS Policlinico Multimedica (A Antonelli); Siracusa, Umberto I, UOC Cardiologia e UTIC (A Marchese); Roma, San Camillo, UOC Cardiologia 1 (M Uguccioni); Cerignola (A Villella); Correggio (A Navazio); Piombino (S Bechi); Roma, Sandro Pertini (F Lo Bianco); San Donato Milanese, IRCCS Policlinico San Donato, UO Cardiologia con UTIC (F Bedogni); Tricase (L Negro); Vizzolo Predabissi (L Donato); Francavilla Fontana (D Statile); Pordenone, Ospedale di Pordenone, SOC Cardiologia (M Cassin); Roma, Umberto Primo, Malattie Cardiovascolari A (F Fedele); Tivoli (A Granatelli); Civitavecchia (S Calcagno); Gravedona (A Politi); Roma, San Pietro FBF (R Serdoz); Cagliari, AO Brotzu, SC Cardiologia (A Pani).
